# Interactions between helminths and tuberculosis infections: Implications for tuberculosis diagnosis and vaccination in Africa

**DOI:** 10.1371/journal.pntd.0008069

**Published:** 2020-06-04

**Authors:** Simeon I. Cadmus, Victor O. Akinseye, Babafemi O. Taiwo, Elena O. Pinelli, Dick van Soolingen, Shelley G. Rhodes

**Affiliations:** 1 Depeartment of Veterinary Public Health & Preventive Medicine, University of Ibadan, Ibadan, Nigeria; 2 Centre for Control and Prevention of Zoonoses, University of Ibadan, Ibadan, Nigeria; 3 Division of Infectious Diseases, Northwestern University Feinberg School of Medicine, Chicago, Illinois, United States of America; 4 Center for Infectious Disease Control Netherlands (CIb), National Institute for Public Health and the Environment (RIVM), Bilthoven, the Netherlands; 5 Department of Medical Microbiology, Radboud University Medical Center Nijmegen, the Netherlands; 6 TB Research Group, Animal and Plant Health Agency, Surrey, United Kingdom; University of Oxford, UNITED KINGDOM

## Abstract

Africa is the second most populous continent and has perennial health challenges. Of the estimated 181 million school aged children in sub-Saharan Africa (SSA), nearly half suffer from ascariasis, trichuriasis, or a combination of these infections. Coupled with these is the problem of tuberculosis (TB) caused by *Mycobacterium tuberculosis* (Mtb) infection, which is a leading cause of death in the region. Compared to the effect of the human immunodeficiency virus on the development of TB, the effect of chronic helminth infections is a neglected area of research, yet helminth infections are as ubiquitous as they are varied and may potentially have profound effects upon host immunity, particularly as it relates to TB infection, diagnosis, and vaccination. Protection against active TB is known to require a clearly delineated T-helper type 1 (Th1) response, while helminths induce a strong opposing Th2 and immune-regulatory host response. This Review highlights the potential challenges of helminth–TB co-infection in Africa and the need for further research.

## Introduction

Africa, with approximately one billion residents, is the second most populous continent and accounts for about 15% of the world's population [1]. As a result of factors beyond the scope of this Review, the continent carries a disproportionate burden of infectious diseases, such as human immunodeficiency virus (HIV), malaria, and tuberculosis (TB) [2]. TB is the leading cause of mortality in sub-Saharan Africa (SSA), with 29% of the 9 million TB cases occurring there in 2013 and 254,000 TB-related deaths [3]. Helminth infections are also highly prevalent with the soil-transmitted helminth (STH) infections, which account for about 85% of the neglected tropical diseases (NTDs) affecting sub-Saharan Africans [4]. Helminth infections are chronic diseases and typically cause asymptomatic infection or prolonged morbidity rather than mortality [5].

Co-endemicity of helminths and other infections in SSA has consequences for public health and affected hosts. Much is already known about the bidirectional interaction of Mtb and HIV; however, there is relatively sparse understanding of the interaction between Mtb and helminth infections—the subject of this Review in the context of Africa—and existing data on the potential immunologic consequences, including those that may affect TB vaccination and diagnosis. This Review supports the need for studies to clarify the impact of helminth co-infection on TB control and how any negative impact might be mitigated, as highlighted by the World Health Organization (WHO) in 2012 in its published top-ten list of research priorities for helminth infections [6].

## Methods

A systematic search was conducted using Google Scholar, Pubmed, CAB Direct, and African Journals Online (AJOL), using the following search words and phrases: helmint*, tubercul*, helmint* and tubercul*, helminth and tuberculosis infection Africa, helminth and tuberculosis diagnos*, and helminth and tuberculosis vaccin*. The review included studies involving helminth, TB, and helminth–TB infection, diagnosis, and vaccination in humans and animals.

## The burden of helminth infection in Africa

Helminths are multicellular worms that belong to three taxonomic groups: cestode (tapeworms), nematode (roundworms), and trematode (flukes). They present a striking variety of life cycles, from direct fecal–oral transmission (ingestion of worm eggs, e.g., of the roundworms *Ascaris lumbricoides* and *Trichuris trichiura*) to development through free-living stages (larval penetration of the skin, e.g., from *Ancylostoma duodenale* hookworm) or dependence on invertebrate vectors (such as the schistosome snail vector). Helminths may also infect via insect bite, for example, from the filarial worms *Onchocerca* (blackfly) and *Brugia* species (mosquito). In SSA, the most common helminth infections are hookworms, followed by schistosomes, ascarids, *Trichuris* whipworms, and lymphatic filariasis (*Brugia*) (**Table 1**) [7–10].

**Table 1:** Disease Burden (DALYs) in SSA Resulting from the NTDs.

Disease	Estimated Global Disease Burden in DALYs	Estimated % Disease Burden in SSA	Estimated SSA Disease Burden in DALYs	References
Hookworm	1.5–22.1 million	34%	0.5–7.5 million	[9 – 11]
Schistosomiasis	1.7–4.5 million	93%	1.6–4.2 million	[8, 10, 12]
Ascariasis	1.8–10.5 million	21%	0.4–2.2 million	[9 – 11]
Lymphatic filariasis	5.8 million	35%	2.0 million	[8]
Trichuriasis	1.8–6.4 million	27%	0.5–1.7 million	[9 –11]
Onchocerciasis	0.5 million	99%	0.5 million	[8]
Total NTDs	More than 49.8 million	15%–37%	5.5 million–16.1 million	[10]

DALY estimates for STH infections and schistosomiasis were obtained by adjusting a wide range of available global estimates according to the percentage of the total number of cases that occur in SSA, while for the other NTDs the disease burdens were quoted directly from WHO estimates. DALY is a WHO measure of overall disease burden expressed as the number of years lost due to ill-health, disability, or early death. Adapted from Hotez and Kamath, 2009 [7]. DALY, disability-adjusted life year; NTD, neglected tropical disease; SSA, sub-Saharan Africa; STH, soil-transmitted helminth; WHO, World Health Organization

In the vast majority of developing tropical and subtropical regions of the world, helminth infections, especially those caused by STHs and schistosomes, constitute major public health challenges, particularly among school aged children who may be nutritionally or physically impaired as a result [10–12]. Current WHO estimates indicate that about 1.5 billion individuals are infected with STH infections globally (https://www.who.int/news-room/fact-sheets/detail/soil-transmitted-helminth-infections), with more than one-half of SSA’s population affected by one or more helminth infections [12,13] Of the estimated 181 million school-aged children in SSA, almost one-half (89 million) are infected with hookworm, ascariasis, trichuriasis, or some combination of these STH infections, which may vary according to factors such as geographical location and socioeconomic status within a given country and even the type of school attended [4, 10–14]. The disability-adjusted life years (DALYs) lost due to these helminth infections provide a more accurate picture of the disease burden, although the estimates of DALYs lost differ greatly from one source to another (Table 1) [15].

The conventional control programs for helminths are based on mass treatment, as recommended by WHO (2017 Guideline: Preventative Chemotherapy to Control STH Infections in At-Risk Population Groups). Regular treatments with broad-spectrum antihelminth drugs (such as the benzimidazoles, mebendazole, or albendazole) are effective at reducing morbidity from STH infections and are well-tolerated, while ivermectin has been employed in areas endemic for filarial diseases. In the case of ivermectin treatment of filariasis in Africa, while progress is being made (e.g., a recent report from Sierra Leone described reductions in *Onchocerca* worm burden [16]), in other countries and areas issues such as poor medicine distribution (e.g., one study in Nigeria [17]) and treatment side effects (e.g, increased epilepsy cases in Tanzania [18]) also need to be addressed if elimination of these debilitating parasites is to be achieved. Even so, a meta-analysis of helminth re-infection studies has shown that prevalence can be quick to re-establish—in this case Ascaris, Trichuris, and hookworms re-established over the ensuing 12 months to 94%, 82%, and 57% of pretreatment levels, respectively [19]. According to the opinion of some experts, treatment of infected individuals, even on a mass scale of drug administration, is not itself sufficient to resolve issues that are fueled by poverty, lack of sanitation, adequate hygiene, and education [20]. Access to a clean water supply to wash fruit and vegetables, identified as an important risk factor particularly in rural areas of Africa [21], could reduce the DALYs lost via such food-borne infection routes [22], supporting the critical role of access to clean water supplies, environmental sanitation, and also education as important to break transmission routes while other potential control measures, such as the use of vaccines are theoretically attractive but remain elusive.

## TB in Africa

TB is a chronic debilitating and wasting disease resulting from infection with Mtb and remains among the leading causes of death from an infectious agent globally. About 5% to 10% of infected humans develop active TB within one year of infection (primary TB). The remainder are classified as individuals with latent TB infection (LTBI). About 5% to 10% of latently infected individuals develop clinical TB during their lifetime via reactivation. Others develop active TB after re-infection with Mtb because LTBI does not provide full immunity against repeated infection [23–24]. Whether or when a latently infected person will develop active TB is summed up by Comstock and colleagues [25]: “Following infection, the incubation period of TB ranges from a few weeks to a lifetime. Both the length and variability of the incubation period are tremendously greater than for nearly all other infectious diseases,” making TB a disease of significant public health importance.

Since the 1990s, TB incidence rates in different parts of the world have developed quite divergently. TB is a major cause of ill health and death, mainly in Africa and Asia where factors like poverty, malnutrition, overcrowding, HIV, poor living conditions, and, of recent, development and spread of multidrug resistant TB are fueling the epidemic. In 2016, the estimated global TB incidence rate was 140 cases per 100,000 persons, which equates to 10.4 million (range of 8.8 to 12.2 million) incident TB cases. Most of the estimated incident TB cases in 2016 were in Southeast Asia (45%) and Africa (25%), with smaller proportions of cases in the Eastern Mediterranean region (7%), Europe (3%), and the Americas (3%) [26]. Whereas in most parts of the world TB incidence rates remained stable or have declined slightly, in Africa, the incidence rates have increased by almost 30%. This increase was most pronounced in the southern half of the continent, especially South Africa, where the incidence nearly quadrupled to approximately 1,000 cases per 100,000 persons.

Bacille Calmette Guerin (BCG), an attenuated strain of *Mycobacterium bovis*, was first used to vaccinate humans against TB in 1921. This vaccine is estimated to provide about 73% protection against fatal forms of childhood TB but shows inconsistent efficacy (with a protection that varies from 0% to 80%) against development and transmission of adult TB [27]. This, coupled with the rising TB epidemic worldwide, compelled WHO to declare TB a global emergency in 1993 [28]. Progress in controlling TB has been slow, and epidemiological models have suggested that global TB elimination targets can only be achieved with a combination of effective TB vaccination, diagnosis, and treatment strategies [29–30]. However, we suggest that included within this strategy must be consideration for those ubiquitous helminth species that have co-evolved with humans and TB and have the potential to undermine TB control by diverting the host immune responses upon which host protection and TB diagnostic tests rely. A better understanding of the effects of helminths on these key interventions may uncover mediating pathways that can be exploited to accelerate the attainment of global TB elimination goals

## Potential impact of helminths on host responses to TB infection, vaccination, and diagnosis

### Containment of Mtb by the immune system

**Fig 1:**
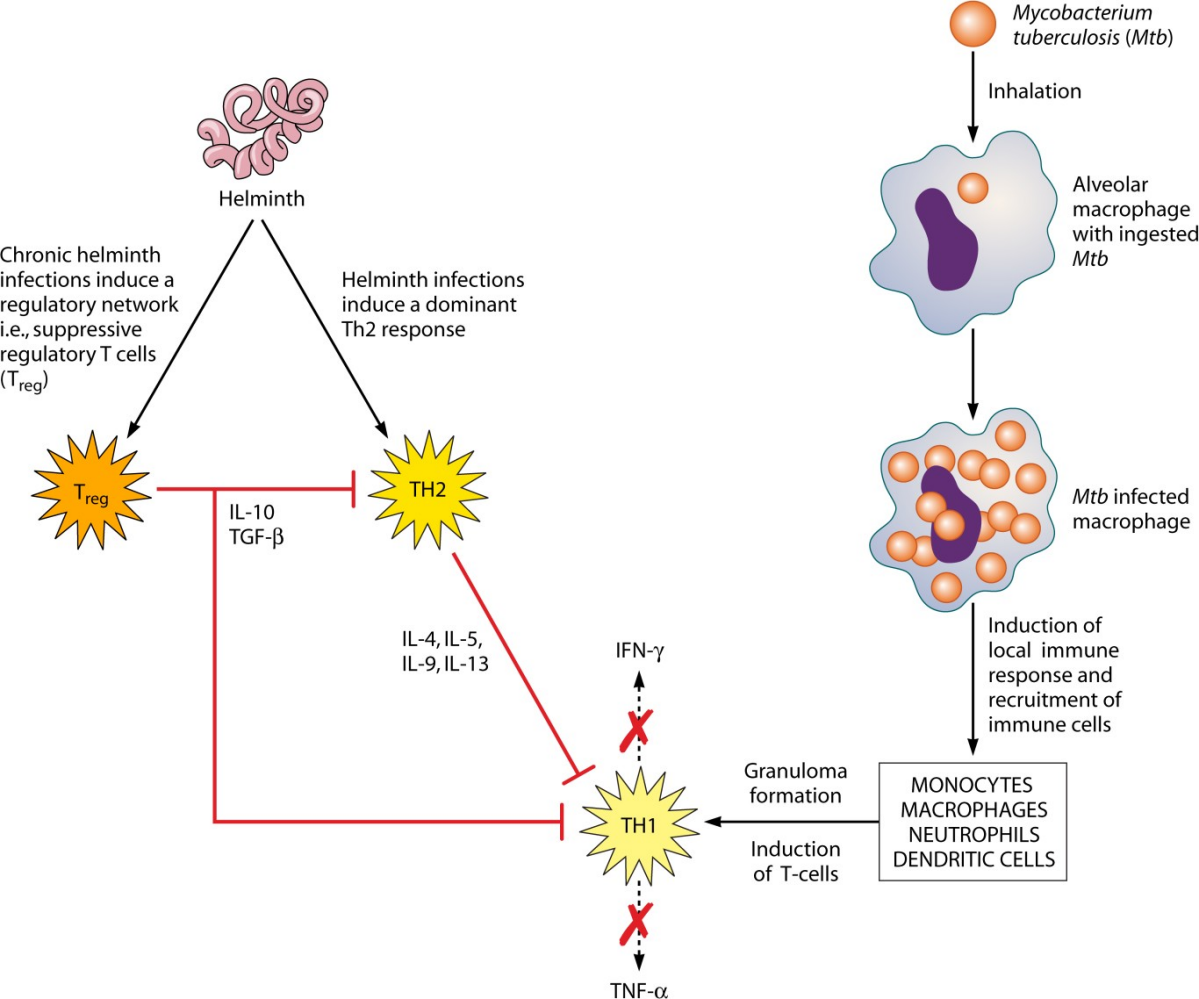
Pathway for the suppression of TH1 by TH2 immune response; a classical scenario of helminth and TB co-infection. IL, interleukin; TH1, T-helper type 1; TGF-β, transforming growth factor β; TNF-α, tumor necrosis factor-alpha.

Upon inhalation of Mtb bacilli, the alveolar macrophages are among the first cells to encounter the micro-organism (Fig 1), making macrophages the first line of defense against invading Mtb. Bacilli become trapped inside a vacuole called phagosome, which thereafter undergoes sequential fusion to acquire microbicidal and degrading characteristics by a process called maturation, which is regulated by the network of Rat sarcoma (Ras) associated binding Guanosine Triphophatases (Rab GTPases), proteins that drive the phagosome progression to maturation [31]. Despite the potential of activated macrophages to kill pathogens, the bacilli can escape this fate and survive within the host macrophage through mediation of pathogen-dependent inhibition of phagosome-lysosome fusion, thus helping it to persist within the immature phagosomal compartment [32]. Further, it was shown that some Mtb strains that were poorly adapted within endocytic vesicles of infected macrophages, through activation of host cytosolic phospholipase A_2_, rapidly escaped from phagosomes and established residence in the cytoplasm of the host cell, thus creating an alternative survival path for Mtb within the host macrophage [33].

Mtb antigen presentation by dendritic cells, potentially aided by neutrophils, in the draining lymph nodes induces a local immune response that results in the recruitment of various cell types to the site of infection, including monocytes, macrophages, neutrophils, and dendritic cells. Together, these cells form a primary granuloma, which, while enclosing the infection, may also permit Mtb growth until T cells are recruited to the infection site (Fig 1) [34]. Protection against active TB is known to require a clearly delineated T-helper type 1 (Th1) response, mediated by interferon-gamma (IFNγ), interleukin-2 (IL2), and tumor necrosis factor-alpha (TNFα) [35–37], which may clear infection or drive it into an immune-mediated containment or latency. Th1 cell responses play a role in the proinflammatory functions necessary for the development of cell-mediated immune responses [38]. IFNγ may also act on cells other than macrophages, and one important function may be to limit polymorphonuclear cell (PMN)-driven inflammation [39]. Mouse models have shown that most susceptible mouse strains exhibit high PMN infiltration in the lungs once infected [40–42], and inhibition of this infiltration improves survival. However, mice that lack IFNγ exhibit high PMN infiltration compared to those lacking cluster of differentiation (CD)4^+^ T cells.

Humans deficient in the production of Th1-type cytokines like IFNγ and its receptor are also known to be susceptible to fulminant mycobacterial infections, including Mtb [42]. IFNγ has been shown to be important in macrophage activation with the resulting stimulation of, for example, nitric oxide synthase and L-arginase production [43–44] and vitamin-D–dependent autophagy, phagolysosome fusion, and bacterial cell death [45].

### Immune responses to helminths and Mtb

Helminths induce a strong Th2 host response that promotes, for example, mucus secretion, collagen deposition, and wound healing mechanisms that are critical for helminth expulsion. Yet despite the induction of such protective Th2 responses, helminths often persist in the host, resulting in chronic infection [46]. Persistence is achieved, in part, by the induction of immunoregulatory pathways that are favorable to helminth survival. Among the immunoregulatory cells induced during chronic helminth infection, regulatory T cells (Tregs) producing cytokines such as transforming growth factor β (TGFβ) and interleukin 10 (IL-10) have been well documented (Fig 1). This expanded population of Tregs can down-modulate both Th1 and Th2 inflammatory responses and interfere with their effector T-cell functions [47–51, 52]. Not surprisingly therefore, prolonged exposure to parasitic helminth infection has been associated with generalized immune hyporesponsiveness [53]. Th2, Tregs, and the immunoregulatory cytokines they produce (such as interleukin-4 [IL4], IL10, and TGFβ) generated during helminth infection may act as potent inhibitors of the Th1 responses which are required for immunity against Mtb infection [54–55] (Fig 1).

### Reactivation of LTBI and severity of active TB

In humans, endogenous reactivation of LTBI has been associated with increased production of IL10 and TGFβ by circulating monocytes and possibly Tregs [56] and also with the inhibition of proinflammatory TNFα, for example, in patients treated with TNFα antagonists for other conditions [57]. Studies have also shown that in patients with LTBI, co-infection with helminths (filariae and hookworms) can induce down regulatory roles on the protective Th1 and Th17 responses required for the control of Mtb infection in LTBI, potentially predisposing towards the development of active disease [58–60]. In the case of filarial infection, this effect may be mediated by both the cytotoxic T lymphocyte antigen (CTLA)–4 and programmed death (PD)–1, with resulting down-regulation of the Th1 proinflammatory cytokines IFNγ, IL-17, IL-12, and IL-23 and restored following antifilarial chemotherapy [59, 61]. However, studies investigating co-existing helminth infection and prevalence of active TB can differ in their conclusions, with one study in Ethiopia showing a doubled risk of active TB in intestinal helminth coinfected individuals [62], while another larger study of intestinal and filarial helminths in India suggested little effect on progression from latent to active pulmonary TB [63].

Experimental rodent models have examined whether helminth co-infection can exacerbate TB pathology. Cotton rats, a natural host for the filarial nematode *Litomosoides sigmodontis*, coinfected with Mtb showed no greater granulomatous inflammation or bacterial burden in the lung when compared to Mtb-only infected animals [64]. Whether this lack of TB exacerbation was due to the induction of regulatory cells and cytokines in this model was not investigated; however, it was noted that the IFNγ responses to tuberculin purified protein derivative (PPD) were the same in Mtb-only and helminth-Mtb coinfected rats. As natural hosts for *L*. *sigmodontis*, it is possible that cotton rats have evolved immune-regulatory pathways that mitigate the effects of this helminth. Overall, the association between helminth infections and immunosuppression is complex since several factors may determine whether infection with the parasite suppresses, exacerbates, or has no effect on immune responses to other infections or unrelated antigens. These factors include the species of the helminth, the parasite load, and whether the host is experiencing a recent or a chronic infection [65].

### BCG immunogenicity

BCG is the most widely used vaccine worldwide and is the only registered vaccine available against TB and leprosy [66–67]. Nevertheless, it has a highly variable efficacy (from 0% to 80%) against pulmonary TB [27], and its protection against this disease has been hypothesized to wane due to gradual attrition of mycobacteria-specific T cells [68]. While BCG vaccination of newborns and infants significantly reduces the risk of childhood complications of TB, because the infant immune system is immature [69–71], it shows a bias towards Th2 cell polarization and low cytokine production, compared with those of adults. BCG vaccination generally induces strong Th1 cell responses, while the degree and quality of these responses vary in different settings [72–74].

Where helminth infection is endemic, particularly in SSA, the newborn is further primed immunologically in utero by its mother to helminth antigens with resultant effect of immunological bias towards Th2 and/or immunoregulatory responses—another route by which helminth infection can ultimately culminate in a reduced immunogenicity of the BCG vaccine [75] and negatively impact upon vaccination programs designed to boost BCG-vaccine-induced immunity. It is known that young children in the United Kingdom and Malawi responded differently to BCG vaccination, with a differential expression of Th1 and Th2 cytokines, respectively, and smaller BCG scars in Malawian infants [76–77]. Whether there was a helminth component acting here was not investigated.

TB vaccines other than BCG are also potentially at risk of helminth-associated reductions in efficacy. The modified vaccinia Ankara virus expressing the immunodominant mycobacterial antigen 85A (MVA85A) is known to induce strong durable Th1 memory cells in adults but only modest cell-mediated immune responses in infants [78–79]. Exploration of the effect of concurrent schistosome infection on the BCG-boosting immunogenicity of the MVA85A vaccine in adolescents [80], however, suggested no detrimental effect of helminth co-infection in these individuals. More recently, vaccine protection against active TB in latently infected adults from South Africa, Kenya, and Zambia has been reported using another subunit vaccine M72/AS01_E_ [81]. It would be nice to think that any further trials to expand this promising data would give some attention to the immune preconditioning that can result from helminth infection.

That a reduced skin test conversion rate following BCG vaccination can result from helminth co-infection was described in 1989 by Kilian and Nielsen [82] in children with onchocerciasis where 45% of helminth-infected children showed vaccine responses, compared to 85% in controls. A later randomized trial showed a lower BCG immunogenicity in individuals with untreated helminth infection compared to infected individuals who received antihelminthic therapy [83]. The reduced responses were associated with a reduced tuberculin PPD-specific interferon gamma (IFN-γ) and IL-12 production and increased PPD-specific TGF-β rather than an increased PPD-specific Th2 profile per se. Similarly, 18-to-24-year-old helminth-infected college students in Ethiopia who were dewormed before BCG vaccination had improved specific immune responses to PPD compared to those who were not dewormed [84].

Immunological imprinting of in utero helminth exposure can affect BCG responses later in life, as has been shown in neonates who received BCG vaccination within 24 hours of birth in rural Kenya, with differing responses to PPD at 10 to 14 months of age; children with in utero sensitization to *Wuchereria bancrofti or Schistosoma haematobium* produced a 26-fold lower PPD-specific IFNγ compared to those whose cord blood lymphocytes showed no evidence of in utero sensitization to these two helminths [85]. However, the antenatal treatment of pregnant women in an area of low helminth prevalence with anthelmintic was shown to have no effect upon the subsequent cellular responses of their children to BCG vaccination [86], suggesting perhaps that the benefit of anthelmintic treatment to BCG vaccine efficacy is mainly to be found where helminth burden is sufficiently high as to interfere with vaccine responses in the first instance. Since much helminth diagnostic testing relies upon low sensitivity methods, like egg counts, this at least provides some confidence that those populations most in need of deworming (high worm infections) would be identifiable using current methods.

Previous studies of revaccination with BCG in order to boost vaccine efficacy had been conflicting, leading WHO to conclude in 1995 that there is no definitive evidence that repeated BCG vaccination confers additional protection against TB [87]. However, a recent trial to assess the efficacy of BCG revaccination versus the candidate TB subunit vaccine H4:IC31 in Cape Town, South Africa [88] has shown both vaccines to reduce transmission (as measured by sustained IFN-γ conversion), with BCG revaccination potentially having the edge. Again, some consideration of preexisting helminth infection as part of such vaccination trials could add value, especially when rolled out to the more rural areas where helminth infection of vaccine recipients might be expected to be higher than in Cape Town.

Exposure to environmental mycobacteria—which like helminths is also considered as higher in rural communities and developing countries—has long been suspected of affecting BCG vaccine efficacy; a review of randomized controlled trials concluded that a higher BCG efficacy was associated with the absence of sensitization with environmental mycobacteria, these geographical areas being further from the equator [89–90]. While meta-analysis has supported a greater BCG vaccine efficacy further away from the equator, it highlighted a number of factors, including environmental mycobacteria, socioeconomic conditions, and nutrition, that could be important [90–91]. Coincidentally, malnutrition may of course be helminth induced. A study of randomly selected adults in Peru showed that protein malnutrition correlated with a higher risk of a false-negative tuberculin skin test result [92]. Even among Canadians, malnutrition was found to be a major risk factor for developing TB [93]. Other possible contributors to the inconsistency of BCG efficacy include (1) genetic or physiological differences among study populations and/or differences in Mtb strains within the populations; (2) strain variation in BCG preparations [94], with strong evidence [95] that genomic differences in BCG strains will ultimately affect the potency of the strain used in a given setting, and (3) the method of preparation itself (e.g., the type of culture medium used to grow vaccine stocks has been shown to influence the later survival of BCG in host cells and the generation of host protective immunity [96]). Overall, then, helminth infection appears to be one of several factors that may compromise the efficacy of BCG vaccination, and this highlights the importance of carefully designed studies to untangle this complex web of observed associations.

### TB diagnosis

Similar to the induction of TB vaccination responses, methods for detecting subclinical TB infection using the tuberculin PPD skin test or the more recent blood IFN-γ tests require a functional host immune response. Thus, modulation of immune responses caused by concurrent helminth infection may reduce the reliability of TB diagnosis. Consistent with this, Stewart and colleagues [97] reported an association between onchocerciasis skin microfilariae density and the down-modulation of cellular responses to PPD plus an age-related skewing of the immune response towards a Th2 profile.

Studies of *M*. *bovis* infection in cattle, a natural host–pathogen relationship, similarly highlight helminth infection as a potential confounder of TB diagnostics. Claridge and colleagues (2014) [98] estimated that exposure to the common fluke *Fasciola hepatica* in dairy herds across England and Wales could be contributing to a reduced diagnosis of *M*. *bovis* infection due to the negative effect of this helminth upon the PPD skin test and a reduced Th1 response in high fluke prevalence areas. However, it was also found that naturally coinfected cattle carried a reduced burden of *M*. *bovis* with an associated suppression of proinflammatory cytokines [99]. Therefore, while helminth co-infection may result in a reduced immune-diagnosis of infected individuals, those individuals could, in fact, pose a lower clinical and TB transmission risk owing to a helminth-driven reduction in bacillary burden. There are clinical co-infection studies that support such a view. Mhimbira and colleagues [100] described a significantly lower sputum bacterial load and lung cavitation among active TB patients in Tanzania coinfected with the blood fluke *S*. *mansoni* compared to those without helminth co-infection. Similarly, Abate and colleagues [101] found that concomitant asymptomatic helminth infection of TB patients in Ethiopia resulted in a lower sputum smear positivity together with an increase in regulatory and Th2 immune responses, a situation that was reversed by antihelminthic drug administration. In both of these studies helminth-driven reductions in sputum bacilli, potentially reflecting an improved host TB infection status (e.g., reduced lung cavitation in Mhimbra and colleagues), at the same time risks a reduction in the sensitivity of TB diagnosis and a subsequent delay in treatment. This would also be a consideration for other tests reliant upon sputum bacillary load such as the XpertMTB/RIF polymerase chain reaction, which has been shown to correlate with sputum smear counts [102].

That helminth-infected individuals might be able to control or modulate their Mtb infection was recently reported in a study of Nepalese immigrants to the UK [103]. In this study, a significant negative association was apparent between hookworm (*Strongyloides*) infection and latent TB (as identified by positive IFN-γ responses). Importantly, this study further demonstrated that blood from hookworm-infected individuals could control the growth of virulent Mtb in vitro, and this control was lost following anthelmintic treatment.

The use of IFNγ release assays (IGRAs) for TB diagnosis has grown in recent years [104–105]. These tests (e.g., QuantiFERON-TB Gold, Cellestis Ltd., Australia and T-SPOT.TB, Oxford Immunotec Ltd., UK) are widely regarded as more sensitive and specific than the skin test and use specific antigens such as ESAT6 and CFP10 that are present in Mtb and *M*. *bovis* but deleted from the *M*. *bovis* BCG vaccine strain [106]. Studies have shown that helminth infection can reduce IFN-γ production in response to mycobacterial infection, for example, above in cattle and in Bangladesh children and pregnant Ethiopian mothers [107–108], risking TB diagnostic sensitivity. However, a recent study of latent TB patients co-infected with hookworm shows that reduced TB-specific IFN-γ responses can be reversed following treatment with anthelmintic [109].

There is evidence to suggest that helminth co-infection does not always have a negative impact on TB immune responses. A study of Amerindians in Venezuela suggested a positive correlation between helminth (*Ascaris* and *Trichuris*) infection and PPD skin test–positivity in household contacts of sputum smear–positive patients [110]. And while a cross sectional study in southern India found no significant association between frequencies of PPD skin test positivity and intestinal helminth or filarial infection, BCG vaccination responses were associated with a lower prevalence of hookworm infection in this study [111]. This highlights the importance of research to understand helminth–TB relationships in a given situation to avoid the danger of assuming that one size will fit all.

### Conflicting issues requiring clarity?

Due to the sometimes-apparent conflicting evidence that helminth co-infection may or may not affect Mtb infection, vaccination, and diagnosis (see Table 2), overall clarity on the consideration of helminth co-infections in TB vaccination and control programs could be timely. Pertinently, at a clinical level, should deworming prior to TB vaccination represent best practice in getting the most effective vaccine response, generating a “window of opportunity” to allow an effective Th1 memory, and for which populations of vaccine recipients (by age, community or area of helminth burden?) and against which specific helminth infections would this be most relevant and beneficial? Indeed, are helminth co-infections a potentially wider issue (i.e., than TB) that could be affecting vaccine success in general [112]?

**Table 2:** Studies implicating and those not supporting helminth co-infection as affecting the diagnosis and outcome of Mtb infections.

S/N	Location of Study/Study type	Helminth(s)	Findings	References
1	*Asia (India) Human Study	Filaria	The investigators revealed that coincident filarial infection exerted a profound inhibitory effect on protective mycobacteria-specific Th1 and Th17 responses in latent tuberculosis, suggesting a mechanism by which concomitant filarial (and other systemic helminth) infections predispose to the development of active TB in humans. They further reported that IFNγ and IL-12 were significantly down-regulated in patients in the PPD+Fil+ group, suggesting that the IL-12/INF- γ pathway in patients with coincident lymphatic filariasis and latent TB was compromised.	[59]
2	**North (USA)	Filaria	The authors indicated that chronic filarial infection does not exacerbate Mtb infection in cotton rat model. They showed that PPD-specific cellular proliferation and IFNγ production were not suppressed in coinfected animals.	[64]
3	*Africa (Ethiopia) Human Study	*Trichuris trichiura*, *Ascaris lumbricoides*, Hookworm, *Tenia spp*, *Hymenolepis nana*, and *Enterobius vermicularis*	Elias and colleagues reported that helminth infections reduced BCG immunogenicity in humans by inducing the production of elevated TGF-β instead of the usual Th2 induced cytokines (IL-4 and IL-5).	[75]
4	*Africa (Ethiopia) Human Study	*Ascaris lumbricoides*, Hookworm, *Strongyloides stercoralis*, *hymenolepis nana*, *Tenia spp*, *Entamoeba histolytica*&*Giardia lamblia*	Elias and colleagues revealed that helminth infections impaired BCG vaccination immunogenicity. However, antihelminthic treatment resulted in enhancement of T-cell proliferation and IFN-γ proliferation with improved BCG efficacy among college students.	[84]
5	*Africa (Kenya) Human Study	Filaria and Schistosoma	Malhotra and colleagues reported that helminth-specific immune responses acquired during gestation persisted into childhood and that this prenatal sensitization biased T-cell immunity induced by BCG vaccination away from Th1 and IFN-γ responses associated with protection against mycobacterial infection.	[85]
6	*Europe (UK)	*Fasciola hepatica*	The authors indicated that *F*. *hepatica* was an additional environmental risk factor for BTB and, importantly, was negatively associated with the odds of BTB being diagnosed on a farm. They suggested that, in the presence of *F*. *hepatica* infection, the SICCT test was less effective.	[98]
7	*Africa (Ethiopia) Human study	*Ascaris lumbricoides*	They found that concomitant asymptomatic helminth infection profoundly affected the immune phenotype of TB patients with a strong leaning towards Th2 types of immune response such as increased regulatory T cells as well as IL-5 and IL-10 secreting cells. Furthermore, helminth co-infection was associated with a significantly lower ratio of sputum smear positivity, which correlated to the egg load in helminth positive TB patients.	[101]
8	*South America (Venezuela) Human Study	*Trichuria trichiura* and *Ascaris lumbricoides*	Here, the authors confirmed that helminth together with low Th1 were associated with TST positivity in pediatric TB contacts.	[110]
9	**Asia (India)	Filaria and Hookworm	Lipner and colleagues showed that neither hookworm nor filarial significantly influenced the delayed-type hypersensitivity response to tuberculin. They reported that BCG vaccination had a protective effect, even in the presence of hookworm and filarial infection.	[109]
10	***Europe (Sweden)	*Hymenolepis diminuta*, *Trichuris muris*, and *Schistosoma mansoni*	Findings revealed that antigens from different species of helminths directly affected macrophage responses to *Mtb*. Antigens from the tapeworm *Hymenolepis diminuta* and the nematode *Trichuris muris* caused an anti-inflammatory response with M2-type polarization and reduced macrophage phagosome maturation and ability to activate T cells, along with increased Mtb burden, especially in *T*. *muris* exposed cells, which also induced the highest IL-10 production upon co-infection. However, antigens from the trematode *Schistosoma mansoni* had the opposite effect causing a decrease in IL-10 production, M1-type polarization, and increased control of *Mtb*.	[111]
11	**North America (New Jersey, USA) Experimental Study	*Nippostrongylus brasiliensis*	Here, Potian and colleagues identified an AAM which was induced via the IL-4Rα signaling pathway in *Nippostrongylus brasiliensis* mouse model. Th2 response in the coinfected mice did not impair the onset and development of the protective Mtb-specific Th1 cellular immune responses. However, the helminth-induced Th2 environment resulted in the accumulation of AAMs in the lung.	[116]
12	* Experimental	*Schistosoma mansoni*	Using a pulmonary mouse model of Mtb infection, the authors demonstrated that *S*. *mansoni* co-infection or immunization with S. mansoni egg antigens can reversibly impair Mtb-specific T cell responses without affecting macrophage-mediated Mtb control. Instead, *S. mansoni* infection resulted in accumulation of high arginase-1–expressing macrophages in the lung, which formed type 2 granulomas and exacerbated inflammation in Mtb-infected mice. Treatment of coinfected animals with an antihelminthic improved Mtb-specific Th1 responses and reduced disease severity.	[117]
13	**New Jersey, USA	*Heligmosomoides polygyrus*, a murine enteric nematode	Rafi and others indicated that prior infection with *Heligmosomoides polygyrus* a murine enteric nematode, did not affect the outcome of primary Mtb infection or challenged infection in vaccinated hosts. Despite the presence of helminth-induced Tregs, resistance to primary Mtb infection was not compromised in coinfected mice.	[120]
14	*Europe (UK) Human study	*Strongyloides* and *Schistosoma*	Helminth infection was associated with a lower frequency of CD4+IFN-γ + T cells, which increased following treatment. Patients with helminth infection showed a significant increase in CD4+FoxP3+ T cells (Treg) compared to those without helminth infection. There was a decrease in the frequency of Treg cells and an associated increase in CD4+IFN-γ + T cells after the anthelmintic treatment. Here, they showed a potential role of Treg cells in reducing the frequency and function of antimycobacterial CD4+IFN-γ + T cells and that these effects were reversed after anthelmintic treatment.	[121]
15	*Asia (India) Human Study	*Wuchereria bancrofti* and *Strongyloides stercoralis*	The authors confirmed that co-existent helminth infection was associated with an IL-10–mediated (for filarial infection) profound inhibition of antigen-specific CD4+ T cell (Th1 &Th17) responses as well as protective systemic cytokine responses in active pulmonary TB. Their study therefore revealed significant alterations in the baseline frequencies of mono—and multifunctional CD4+ and CD8 + Th1 and Th17 cells in TB-infected individuals with active helminth infection.	[122]
16	*Asia (India)	Hookworm	The authors revealed that coincident hookworm infection exerted a profound inhibitory effect on protective Th1 and Th17 responses in latent TB and therefore predisposed toward the development of active TB in humans.	[123]
17	*Europe (Sweden) Experimental	*Schistosoma mansoni*	Elias and others confirmed that *S*. *mansoni* infection reduced the protective efficacy of BCG vaccination against Mtb possibly by attenuation of protective immune responses to mycobacterial antigens and/or by polarizing the general immune responses to the Th2 profile in mice.	[124]
18	*South America (Brazil)	*Toxocara canis* and *Schistosoma mansoni*,	Frantz and colleagues demonstrated that the therapeutic effects of DNAhsp65 (a DNA vaccine that codifies heat shock protein Hsp65 from *M*. *leprae*, which is used in therapy during experimental TB) in experimental TB infection was persistent in the presence of an unrelated Th2 immune response induced by helminth infections in mice.	[125]
19	*Europe *(*Sweden*)*	*Schistosoma mansoni*	The authors indicated that *S*. *mansoni* coinfected mice had significantly higher levels of BCG bacilli in their organs and sustained greater lung pathology compared *to Schistosoma* uninfected controls.	[126]
20	*Europe (Ireland)	*Fasciola hepatica*	Flynn and colleagues demonstrated that *F*. *hepatica* altered irresponsiveness (delayed-type hypersensitivity reaction and cytokine responses) to virulent *M*. *bovis*, thus inducing the reduction of IFN-γ responsiveness in coinfected animals.	[127]
21	*Europe (Ireland)	*Fasciola hepatica*	Flynn and colleagues found the predictive capacity of tests (SCITT and the IFN-γ) to be compromised in coinfected animals and that *F*. *hepatica* infection altered macrophage function. IL-4 and IFN-γ expression in whole-blood lymphocytes restimulated in vitro with *M*. *bovis* antigen was also altered in coinfected animals. These results raised the question of whether *F*. *hepatica* infection can affect the predictive capacity of tests for the diagnosis of BTB and possibly also influence susceptibility to BTB and other bacterial diseases.	[128]

AAM, alternatively activated macrophage; BCG, Bacille Calmette Guerin; BTB, bovine tuberculosis; CD, cluster of differentiation; IFN-γ, interferon gamma; Mtb, *Mycobacterium tuberculosis*; PPD, purified protein derivative; SCITT, single cervical intradermal tuberculin test; TB, tuberculosis; TGF-β, transforming growth factor β; Th1, T-helper type 1; Treg, regulatory t cells; TST, tuberculin skin test

*In agreement

** Not in agreement

*** Indicated both agreement and not in agreement

At a more fundamental research level, the conflicting scenarios presented by different studies may not be unrelated to the fact that antigens from different helminth species cause different responses towards Mtb within host macrophages [113]—with the effect of either enhancing or diminishing the bactericidal function of macrophages and potentially priming the downstream adaptive immune response, such as the recently described DNA methylation of CD4+ T cells that appears to be helminth-specific [114]. Based upon these, important initial interactions could result an increased mycobacterial burden [115–116], a reduction [116–118], or have no effect [119]. Therefore helminth-driven skewing of the earliest immune responses to mycobacterial infection in the long term could provide novel interventions of immense potential future value.

## Conclusion

Immunity to TB depends upon a protective Th1 response, which may be subverted by parasitic helminths that are biased towards inducing opposing Th2 responses. Furthermore, the chronic nature of helminth infections also invoke immune-regulatory responses that can reduce TB immunity and interfere with induced diagnostic responses upon which TB control programs depend. Since there is widespread helminth co-infection in areas of high TB incidence in Africa, the immunomodulation engendered by these common but different helminth infections may be a critical determinant for host immunity to TB, diagnostic tests, and the efficacy of preventive vaccines. The impact of these co-evolved microbial and parasitic interactions on the strategies required for optimal public health would appear daunting, particularly considering the apparent complexity and conflicting outcomes in disparate helminth–TB co-infection studies. However, there is the tantalizing potential for improvement in our diagnostic and vaccination outcomes by addressing this relatively neglected component of host TB immunology.

**Key learning points**

- Africa is characterized by plethora of problems including poverty, poor hygiene, and sanitation, which are exacerbated by infectious diseases and weak health systems. Unfortunately, these problems are also worsened by an overlapping burden of helminth and TB co-infection with far reaching public health implications, though currently attracting little attention.
- There are contradictory reports of differences in the population responses to BCG vaccination. However, reports abound that helminth-specific immune responses acquired during gestation persisted into childhood. Thus, the prenatal sensitization induced by helminths, biases the T cell immunity away from Th1 IFN-γ responses associated with protection against mycobacterial infection.
- There are existing reports on the negative impact of helminthes on TB diagnosis. This becomes important where asymptomatic helminth infection profoundly affects the immune phenotype of TB patients with a strong bias towards Th2 types of immune response, such as increased regulatory T cells as well as IL-5 and IL-10 secreting cells.
- There are increasing challenges that helminth–TB co-infection are also found to be associated with a significantly lower ratio of sputum smear positivity, which correlates with the egg load in helminth positive TB patients. This is of great public health importance in areas of the world, particularly Africa, where the burden of TB is high coupled with poor diagnostic facilities.
- The association between helminth infections and immunosuppression is complex since several factors may determine whether infection with the parasite suppresses, exacerbates, or has no effect on immune responses towards TB. These includes the species of helminths, the parasite load, and whether the human host is experiencing a recent or a chronic infection. These are critical issues that need further investigations in resolving the complex challenges posed by helminth–TB co-infection regarding diagnosis, treatment, and vaccination strategies in tuberculosis. The prevalent helminth co-infection in areas of high TB incidence in Africa remains an important factor that will determine the immunomodulation caused by the common but different helminth infections towards host immunity to TB, diagnostic tests, and the efficacy of preventive vaccines. Moving forward, it will be important to confirm if prior deworming to TB vaccination imply best practice in achieving optimal vaccine response, and for which helminth infections and human population groups would this be useful?

**Top five papers**

1. Babu S, Bhat SQ, Kumar NP, Jayantasri S, Rukmani S, Kumaran P et al. Human type 1 and 17 responses in latent tuberculosis are modulated by coincident filarial infection through cytotoxic T lymphocyte antigen–4 and programmed death–1. J Infect Dis. 2009; 200:288–298.
2. Elias D, Britton S, Aseffa A, Engers H, Akuffo H. Poor immunogenicity of BCG in helminth infected population is associated with increased in vitro TGF-beta production. Vaccine. 2008; 26(31):3897–902. doi: 10.1016/j.vaccine.2008.04.083.
3. Aira N, Andersson AM, Singh SK, McKay DM, Blomgran R. Species dependent impact of helminth-derived antigens on human macrophages infected with *Mycobacteriumtuberculosis*: Direct effect on the innate antimycobacterial response. PLoS Negl Trop Dis. 2017; 11(2): e0005390.doi:10.1371/journal. pntd.0005390.
4. Abate E, Belayneh M, Idh J, Diro E, Elias D, Britton S. et al. Asymptomatic helminth infection in active tuberculosis is associated with increased regulatory and Th-2 responses and a lower sputum smear positivity. PLoS Negl Trop Dis. 2015; 9(8): e0003994. 10.1371/journal.pntd.0003994.
5. Potian JA, Rafi W, Bhatt K, McBride A, Gause WC, Salgame P. Preexisting helminth infection induces inhibition of innate pulmonary anti-tuberculosis defense by engaging the IL-4 receptor pathway. J Exp Med. 2011; 208: 1863±74. doi: 10.1084/jem.20091473.
